# Effects of Glass Fiber Content on the Cellular Morphology and Mechanical Properties of Microcellular Injection-Molded Polypropylene Composite Foams

**DOI:** 10.3390/polym18172089

**Published:** 2026-08-28

**Authors:** He Zhang, Wei Guo

**Affiliations:** 1 State Key Laboratory of Light Superalloys, Wuhan University of Technology, Wuhan 430070, China; 13553031189@163.com; 2 Hubei Key Laboratory of Advanced Technology for Automotive Components, Wuhan University of Technology, Wuhan 430070, China; 3 Hubei Collaborative Innovation Center for Automotive Components Technology, Wuhan University of Technology, Wuhan 430070, China; 4 Hubei Research Center for New Energy & Intelligent Connected Vehicle, Wuhan University of Technology, Wuhan 430070, China

**Keywords:** glass-fiber-reinforced polypropylene, MuCell injection molding, cellular morphology, cell refinement–foam expansion balance, specific mechanical properties

## Abstract

Glass-fiber-reinforced polypropylene (GF/PP) composite foams combine reinforcement with weight reduction, but how GF content governs the competition between cell refinement and foam expansion under fixed-cavity molding remains unclear. GF/PP composite foams containing 0–15 wt.% GF and a fixed 5 wt.% PP-g-MAH were prepared by fixed-cavity MuCell injection molding. Gas-free melt rheology, crystallization, cellular morphology, density, and mechanical properties were evaluated. Increasing GF content raised the apparent viscosity and shifted crystallization toward higher temperatures. The cell density increased from 6.12 × 10^4^ to 1.56 × 10^7^ cells cm^−3^, whereas the average cell diameter decreased from 264.5 to 27.1 μm. However, the expansion ratio peaked at 1.27 for GF9 and then decreased to 1.12 for GF15, showing that continuous cell refinement did not produce a monotonic improvement in foam expansion. These trends suggest competing effects of GF on cellular development: at low-to-intermediate contents, GF interfaces may favor heterogeneous nucleation, while increased melt resistance may suppress excessive cell growth and coalescence; at higher contents, fiber crowding, reduced interfiber spacing, geometric confinement, and interfacial heterogeneity may increasingly restrict cell growth and foam expansion. Although the absolute tensile and flexural properties increased with GF content, the density-normalized properties and impact strength varied nonmonotonically. GF9 exhibited the highest measured mean specific tensile, flexural, and impact strengths, together with a mean impact strength of 24.4 kJ·m^−2^, which was 32.6% higher than that of GF0. Thus, cell refinement alone does not ensure improved lightweight efficiency; an intermediate GF content provides the most favorable balance among cellular refinement, foam expansion, and fiber reinforcement.

## 1. Introduction

Automotive lightweighting is an important strategy for improving fuel economy, extending the driving range of electric vehicles, and reducing CO_2_ emissions [1]. Glass-fiber-reinforced polypropylene (GF/PP) composite foams combine the mechanical reinforcement provided by GF with the weight reduction achieved through foaming, making them promising materials for automotive applications such as interior and exterior trim, instrument-panel carriers, door modules, pillar trim, seat supports, under-hood covers, and bumper energy-absorbing structures [2,3,4].

Supercritical-fluid-assisted microcellular injection molding using N_2_ or CO_2_ as the blowing agent enables the efficient, high-volume production of automotive components made from GF/PP composite foams [5,6,7]. Although GF provides high strength and stiffness, its density is higher than that of PP. Excessive GF addition not only increases the density of the foamed composites and thereby compromises the weight reduction of automotive components, but also reduces melt flowability and restricts cell growth. Therefore, achieving an appropriate balance between fiber reinforcement and cellular weight reduction remains a key challenge in the microcellular injection molding of GF/PP composite foams.

Classical nucleation theory predicts that solid additives can promote heterogeneous cell nucleation by reducing the free-energy barrier at the polymer/additive interface; however, the nucleation efficiency depends on the additive concentration, interfacial energy, wetting behavior, and gas-saturation conditions [8]. Studies on injection-molded PP foams have further demonstrated that the final cellular morphology is governed not only by the availability of nucleation sites but also by matrix rheology, gas content, pressure history, and cooling conditions [9]. Xi et al. [10] investigated scN_2_-assisted microcellular injection molding of GF/PP composites at reported mass ratios of 11.8%, 21.8%, and 29.6%. Under their optimized conditions at the lowest GF level, an average cell size below 30 μm and a weight reduction of approximately 10.5% were achieved. The foamed specimens exhibited tensile and flexural strengths comparable to those of their solid counterparts, whereas the impact strength increased from 50.74 to 76.44 J m^−1^. However, increasing the GF content resulted in less uniform fiber dispersion and orientation, and the impact strength did not increase monotonically.

Gómez-Monterde et al. [11] studied a chemically coupled PP composite containing a fixed GF content of 20 wt.% using MuCell injection molding. The resulting foams exhibited cell sizes of approximately 2–252 μm and cell densities of 1.0 × 10^6^–5.5 × 10^6^ cells cm^−3^, depending on the sampling position and weight-reduction level. Although the absolute flexural and impact properties decreased with apparent density, the corresponding specific properties were largely maintained. Micro-CT measurements also revealed an average residual fiber length of 740 ± 150 μm and position-dependent fiber orientation. Gómez-Monterde et al. [12] extended the investigation to long-glass-fiber-reinforced PP foams produced using MuCell and Ku-Fizz foaming technologies, with or without Core Back cavity expansion. The SEM and micro-CT analyses revealed characteristic skin-core cellular structures, with cell sizes ranging from 5 to 248 μm and cell densities on the order of 10^6^ cells cm^−3^. Although the absolute tensile, flexural, and impact properties generally decreased with decreasing foam density, the reinforcement and preferential alignment of the long glass fibers helped maintain the specific mechanical properties. These results demonstrate that the cellular morphology and mechanical performance of GF/PP composite foams depend strongly on the combined effects of fiber reinforcement and the selected foaming route. Yang et al. [13] examined GF/PP composite foams containing 0–20 wt.% GF produced by N_2_-assisted mold-opening microcellular injection molding. At a mold-opening distance of 4 mm, adding 5 wt.% GF increased the cell size from 32.40 to 152.77 μm and decreased the cell density from 1.23 × 10^9^ cells cm^−3^ to 9.04 × 10^7^ cells cm^−3^, whereas further increasing the GF content to 20 wt.% reduced the cell size to 79.15 μm and increased the cell density to 3.85 × 10^8^ cells cm^−3^. These results demonstrate that the effect of GF content on cellular morphology is process-dependent and not necessarily monotonic.

To facilitate a quantitative comparison, Table 1 summarizes the GF content, processing conditions, cell size, cell density, and representative mechanical properties reported in selected studies on GF/PP composite foams. Because specimen geometry, foam density, sampling position, and testing conditions differed among these studies, the listed data are intended to illustrate general trends rather than provide a direct performance ranking.

Beyond its influence on cellular morphology and mechanical performance, GF also affects the crystallization behavior of the PP matrix. Evidence from non-foamed GF/PP composites can therefore provide complementary information on GF-induced crystal nucleation and restricted crystal growth. Iroh and Berry [14] reported that 30 wt.% short GF increased the peak crystallization temperature of PP from 371.0 to 382.5 K and increased the crystallization rate by approximately two orders of magnitude relative to neat PP. Wang et al. [15] subsequently found that the crystallinity of injection-molded GF/PP composites increased from 41.4% for neat PP to 45.7% at 10 wt.% GF, but decreased to 32.9% at 30 wt.% GF, indicating that GF-assisted crystal nucleation and restricted crystal growth may coexist. These studies concern crystal nucleation in non-foamed composites and should not be interpreted as direct evidence of cell-nucleation kinetics.

Collectively, previous studies have demonstrated that GF can affect cell nucleation, melt rheology, crystallization, fiber orientation, and the mechanical performance of GF/PP composite foams. However, it remains unclear how low-to-moderate short-GF contents affect the balance among cell refinement, foam expansion, density reduction, and mechanical reinforcement under identical fixed-cavity MuCell processing conditions. Mold-opening experiments couple the effect of GF content with changes in cavity volume and expansion ratio, making it difficult to distinguish the influence of GF content under fixed-cavity conditions. Therefore, the present study systematically varies the short-GF content from 0 to 15 wt.% in increments of 3 wt.% while maintaining PP-g-MAH at 5 wt.% of the total formulation. The rheological behavior of melts without dissolved gas, crystallization behavior, cellular morphology, apparent density, and both absolute and specific mechanical properties are evaluated under otherwise identical processing conditions. These measurements are used to determine whether an intermediate GF content provides a favorable balance between foam-induced weight reduction and fiber reinforcement. The resulting trends are discussed in terms of a possible competition between cell nucleation and growth; no direct measurement of these kinetics is claimed.

## 2. Materials and Methods

### 2.1. Materials

Polypropylene (PP, grade K8303), with a density of 0.90 g cm^−3^ and a melt mass flow rate (MFR) of 2.0 g/10 min measured at 230 °C under a load of 2.16 kg, was supplied by SINOPEC Beijing Yanshan Petrochemical Co., Ltd., Beijing, China. Maleic anhydride-grafted polypropylene (PP-g-MAH, grade CMG9801), with a maleic anhydride grafting degree of 0.98%, was provided by Fine-Blend Polymer (Shanghai) Co., Ltd., Shanghai, China. Chopped glass fibers (GFs, grade ECS301T), with a nominal filament diameter of 12–14 μm and a nominal chopped length of 3 mm, were supplied by Chongqing Polycomp International Corporation (CPIC), Chongqing, China. Nitrogen (N_2_, purity > 99%) was supplied by Wuhan Xiangyun Gas Co., Ltd., Wuhan, China.

### 2.2. Preparation Process of GF/PP Composite Foams

PP and PP-g-MAH were dried in a forced-air drying oven (101-3FD, Shanghai Huyueming Scientific Instrument Co., Ltd., Shanghai, China) at 70 °C for 6 h. The dried materials and chopped glass fibers were premixed according to the formulations listed in Table 2 using a vertical mixer (KHS-100, Huizhou Taiqiang Plastic Machinery Co., Ltd., Huizhou, China). The resulting mixtures were subsequently melt-compounded and pelletized using a twin-screw extruder (SHJ-20, Nanjing GIANT Machinery Co., Ltd., Nanjing, China) at a screw speed of 60 r/min. The barrel temperature profile from the feed zone to the die was set to 160, 180, 190, 200, and 200 °C.

The injection-molded foamed specimens were prepared using an injection-molding machine (HDX50, Ningbo Haida Plastic Machinery Co., Ltd., Ningbo, China) equipped with a MuCell^®^ supercritical-fluid foaming system (T-100, Trexel, Inc., Wilmington, MA, USA). The N_2_ dosing pressure and dosing duration of the MuCell^®^ system were 180 bar and 3 s, respectively. The barrel temperature profile of the injection-molding machine was set to 190, 200, 200, and 200 °C. The melt injection pressure, injection time, and cooling time were 50 MPa, 1 s, and 50 s, respectively. The resulting specimens included tensile test specimens (150 mm × 10 mm × 4 mm), flexural test specimens (80 mm × 10 mm × 4 mm), and V-notched Izod impact test specimens (80 mm × 10 mm × 4 mm).

### 2.3. Testing and Characterization

#### 2.3.1. Rheological Testing

The rheological behavior of the samples was characterized using a capillary rheometer (CR-6000, Gotech Testing Machines (Dongguan) Co., Ltd., Dongguan, China). Approximately 15 g of each sample was loaded into the rheometer barrel and heated to 200 °C. The plunger speed and acceleration were set to 0.1 mm s^−1^ and 1.2 cm s^−2^, respectively, and the corresponding shear rate and apparent shear viscosity were recorded.

#### 2.3.2. Thermal Analysis

The thermal behavior of the samples was characterized using a differential scanning calorimeter (DSC 214, NETZSCH-Gerätebau GmbH, Selb, Germany). Approximately 10 mg of each sample was weighed and placed in an aluminum pan. The sample was first heated from approximately 25 to 230 °C at a heating rate of 10 °C min^−1^ and held at 230 °C for 5 min to eliminate its previous thermal history. It was then cooled to approximately 25 °C at a cooling rate of 20 °C min^−1^ and subsequently reheated to 230 °C under the same heating conditions. The cooling and second-heating curves were used for analysis. The crystallinity ($\chi_{c}$) was calculated as follows:

(1) $$\chi_{c}=\frac{{\Delta H}_{m}}{{\Delta H}_{0}\times \left(1-\varphi_{GF}\right)}\times 100\%$$

where ${\Delta H}_{m}$ is the melting enthalpy measured for the composite; ${\Delta H}_{0}$ is the melting enthalpy of 100% crystalline PP (209 J g^−1^); and $\varphi_{GF}$ is the mass fraction of GF in the composite. Accordingly, (1 − $\varphi_{GF}$) equals the combined mass fraction of PP and PP-g-MAH. Because PP-g-MAH has a PP backbone and was fixed at 5 wt.% in all formulations, it was treated as part of the PP-based polymer phase for this normalization. Therefore, $\chi_{c}$ should be interpreted as the apparent crystallinity of the PP-based polymer phase rather than the absolute crystallinity of neat PP.

#### 2.3.3. Morphological Characterization

The microcellular morphology of the GF/PP composite foams was observed using a scanning electron microscope (JSM-IT300, JEOL Ltd., Tokyo, Japan). The average cell diameter ($\bar{D}$) and cell density (N) were calculated using Equations (2) and (3), respectively:

(2) $$\bar{D}=\frac{1}{n}\sum_{i=1}^{n}D_{i}$$

(3) $$N={\left(\frac{n}{A}\right)}^{\frac{3}{2}}$$

where $D_{i}$ is the diameter of the *i*-th cell, n is the total number of cells identified within the analyzed region, and A is the calibrated area of that region. At least three independently prepared specimens were examined for each formulation, and SEM images were acquired at three different locations on each specimen. All acquired images were included in the quantitative analysis, whereas only representative images are presented in the figures.

The expansion ratio (ER) of the GF/PP composite foams was calculated using Equation (4):

(4) $$ER=\frac{\rho_{u}}{\rho_{f}}$$

where $\rho_{u}$ and $\rho_{f}$ are the densities of the corresponding unfoamed and foamed composites, respectively.

#### 2.3.4. Mechanical Testing

The tensile and flexural properties of the specimens were measured using an electronic universal testing machine (CMT6104, MTS Systems (China) Co., Ltd., Shanghai, China). The notched Izod impact strength was measured using an impact tester (XJUD-5.5, Chengde Jinjian Testing Instrument Co., Ltd., Chengde, China) with an impact energy of 2.75 J. For each formulation, ten valid measurements were obtained for each mechanical property. The results are reported as the mean values, and the error bars represent the corresponding standard deviations.

The density-normalized specific mechanical properties were calculated using Equation (5):

(5) $$P_{s}=\frac{P_{i}}{p_{f}}\;$$

where $P_{i}$ denotes the measured tensile strength, flexural strength, or notched impact strength, and $P_{s}$ denotes the corresponding specific tensile strength, specific flexural strength, or specific impact strength.

## 3. Results and Discussion

### 3.1. Rheological Behavior

**Figure 1:**
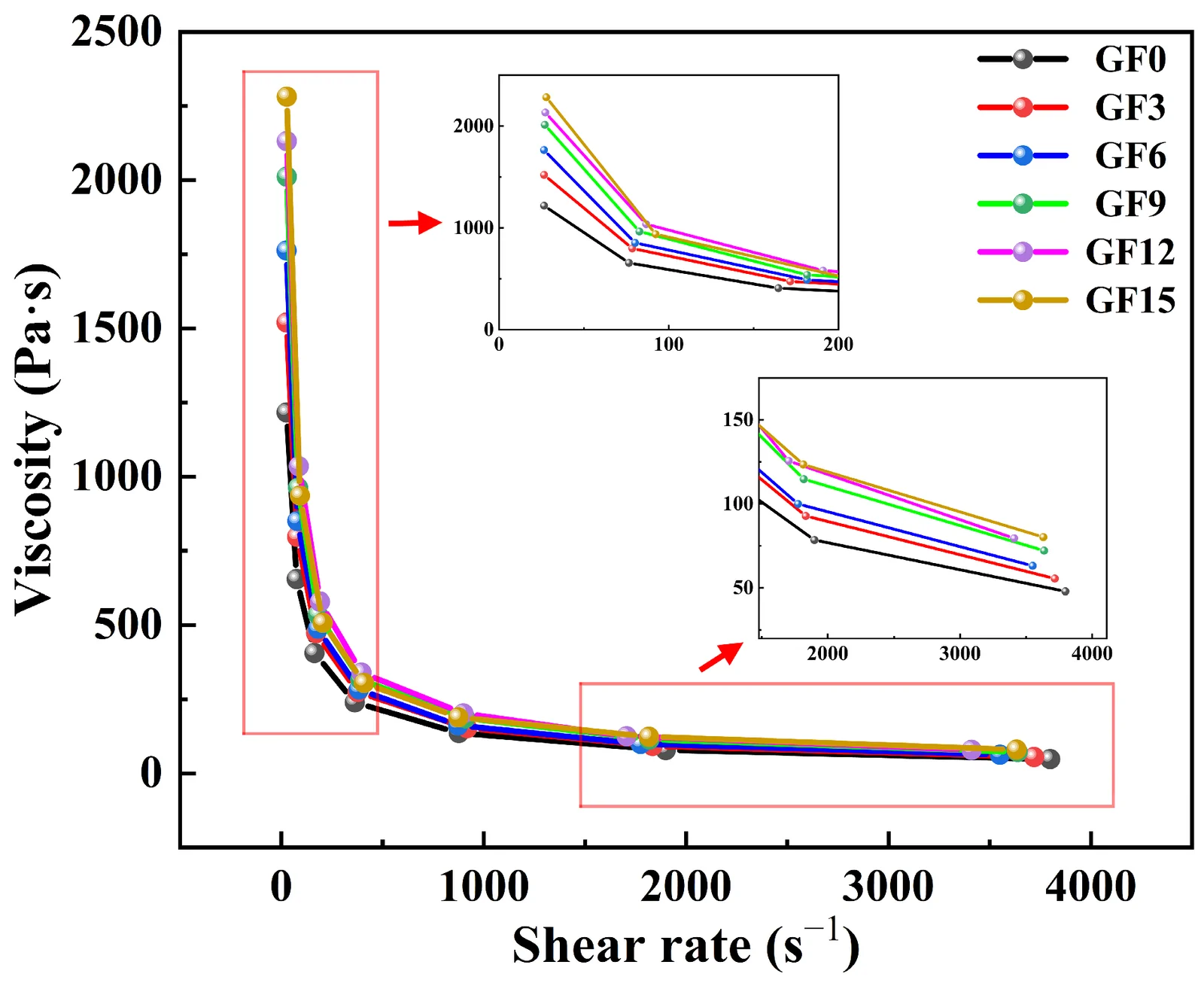
Apparent shear viscosity as a function of shear rate for GF/PP composites with different GF contents.

Figure 1 shows the apparent shear viscosity as a function of shear rate for GF/PP composites with different GF contents. All samples exhibited typical shear-thinning behavior, with the apparent viscosity decreasing as the shear rate increased, indicating the pseudoplastic nature of the composite melts. This behavior is mainly attributed to the progressive disentanglement and alignment of PP molecular chains under shear. The fibers may also increasingly orient in the flow direction, thereby reducing their resistance to melt flow.

Compared with GF0, the GF-containing composites generally exhibited higher apparent viscosities, particularly at low shear rates. The rigid fibers restrict PP-chain mobility and increase the hydrodynamic resistance of the melt. Fiber–fiber interactions may also become more pronounced with increasing GF content, resulting in higher low-shear-rate viscosities. GF15 exhibited the highest viscosity in this region, indicating the greatest resistance to shear deformation. At higher shear rates, the viscosity differences among the formulations gradually decreased, possibly because of enhanced molecular-chain alignment and the increasing tendency of the fibers to orient along the flow direction. Nevertheless, GF15 maintained a higher apparent viscosity than GF0 throughout the investigated shear-rate range.

The capillary rheometric results describe only the baseline behavior of gas-free melts under steady shear and do not represent the actual rheological state of the N_2_-laden melt during microcellular injection molding. Under foaming conditions, melt flow and cellular morphology are additionally affected by N_2_-induced plasticization, cavity-pressure evolution, gas diffusion, cell growth, and rapid melt solidification. Because neither the viscosity of the N_2_-laden melts nor the cavity-pressure profiles were measured, the actual melt viscosity and cell-development kinetics cannot be quantified here. The gas-free data nevertheless show that adding GF increases resistance to melt deformation. This effect may oppose the additional heterogeneous nucleation sites provided by the GF surfaces, especially at high GF contents.

### 3.2. Crystallization Behavior

**Figure 2:**
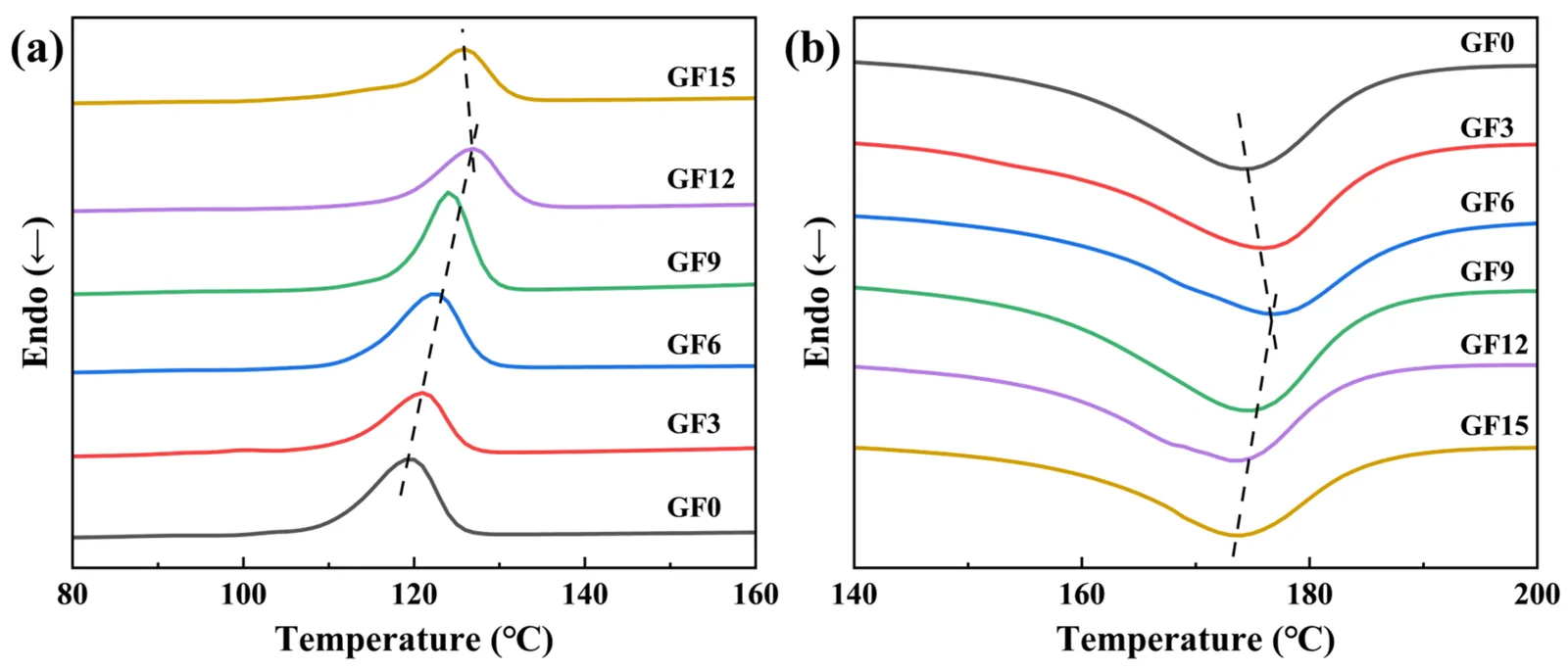
DSC curves of GF/PP composites with different GF contents: (**a**) cooling crystallization curves and (**b**) second-heating melting curves.

Figure 2 presents the DSC cooling crystallization and second-heating melting curves of the GF/PP composites, and the corresponding thermal parameters are summarized in Table 3. For GF0, the peak crystallization temperature (*T*_c_) and onset crystallization temperature (*T*_onset_) were 119.6 and 125.0 °C, respectively. With increasing GF content, both temperatures generally shifted toward higher values and reached their maxima of 126.9 and 132.8 °C, respectively, for GF12. A slight decrease was subsequently observed for GF15, for which *T*_c_ and *T*_onset_ were 125.9 and 131.4 °C, respectively. The shifts toward higher temperatures indicate that GF facilitates the crystallization of the PP matrix. This behavior is consistent with heterogeneous crystal nucleation associated with the GF surfaces, which may enable PP chains to initiate crystallization at higher temperatures [14,15].

The apparent crystallinity (*χ*_c_) increased from 27.7% for GF0 to 29.7% and 30.2% for GF3 and GF6, respectively. After reaching its maximum value at 6 wt.% GF, *χ*_c_ decreased to 28.5%, 27.5%, and 27.2% for GF9, GF12, and GF15, respectively. The maximum at GF6 indicates that a moderate GF addition favored crystal formation, whereas further addition probably restricted PP-chain rearrangement and crystal growth. In addition, because PP-g-MAH was maintained at 5 wt.% while the GF content varied, the resulting change in the PP-g-MAH/GF ratio may also influence the interfacial environment and crystallization behavior.

The melting temperatures of the composites ranged from 173.6 to 176.8 °C and showed no monotonic dependence on GF content. These results indicate that GF content has a more pronounced influence on the crystallization process of the PP matrix than on the melting temperature of the resulting crystals.

### 3.3. Microcellular Morphology

**Figure 3:**
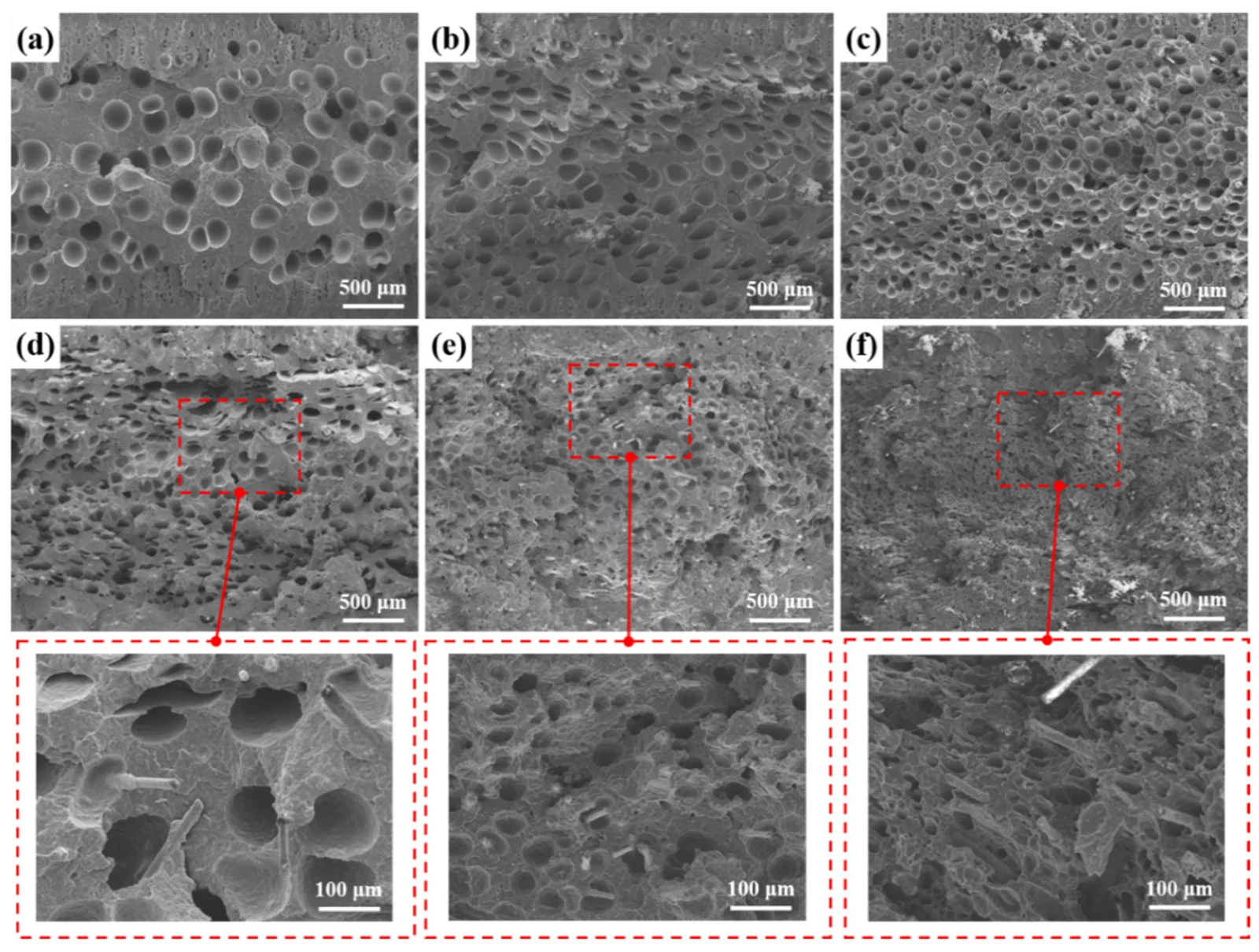
SEM micrographs of GF/PP composite foams: (**a**) GF0; (**b**) GF3; (**c**) GF6; (**d**) GF9; (**e**) GF12; and (**f**) GF15.

**Figure 4:**
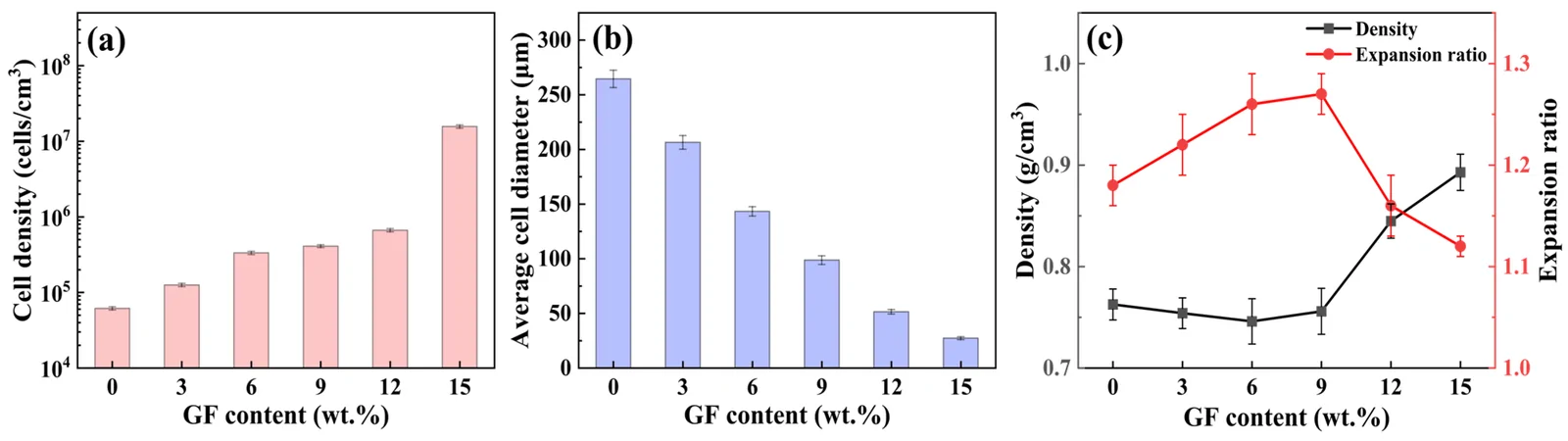
Cellular morphology and density-related parameters of GF/PP composite foams: (**a**) cell density; (**b**) average cell diameter; and (**c**) foam density and expansion ratio. Error bars represent standard deviations from at least three independently prepared specimens.

Figure 3 presents cross-sectional SEM images of the GF/PP composite foams, and Figure 4 summarizes the corresponding cellular parameters. The GF0 sample exhibited relatively large and nonuniform cells, particularly in the core region. With increasing GF content, the cells became progressively smaller and more numerous. From GF0 to GF15, the cell density increased from 6.12 × 10^4^ cells cm^−3^ to 1.56 × 10^7^ cells cm^−3^, while the average cell diameter decreased from 264.5 to 27.1 μm. These trends are consistent with heterogeneous cell nucleation at the GF/polymer interfaces. As the number of nucleated cells increases, individual cells compete more strongly for the available gas and growth space, thereby contributing to the reduction in cell size.

The enlarged images in Figure 3d–f show that some fibers were embedded in the cell walls or locally extended across cell cavities, indicating a close spatial interaction between the fibers and the developing cells. The rigid fibers may locally restrict cell expansion, particularly as the interfiber spacing decreases at higher GF contents. Meanwhile, cell development is also affected by the plasticization of the melt by supercritical N_2_, gas availability, pressure evolution, and cooling and solidification. The final cellular morphology therefore results from the combined effects of heterogeneous nucleation and subsequent cell-growth conditions.

Figure 4c shows that the expansion ratio increased from 1.18 for GF0 to 1.27 for GF9. Over the same composition range, the foam density remained relatively low and reached a minimum of 0.746 g cm^−3^ for GF6. When the GF content increased to 12 and 15 wt.%, the expansion ratio decreased to 1.16 and 1.12, respectively, while the corresponding foam density increased to 0.845 and 0.893 g/cm^3^. Although the higher intrinsic density of GF increases the density of the solid composite, this contribution is initially offset by the cellular structure. At higher GF contents, however, the reduction in expansion ratio weakens the density-reduction effect of foaming.

The lower expansion ratio at high GF contents may be associated with the increased resistance of the composite melt to deformation, reduced interfiber spacing, and greater local fiber crowding, all of which can restrict the space available for cell expansion. In addition, because PP-g-MAH was fixed at 5 wt.% of the total formulation, the amount of compatibilizer relative to GF decreased with increasing GF content and may have influenced fiber dispersion and the local interfacial environment. Thus, increasing the GF content continuously refined the cells but did not continuously improve foam expansion. The GF9 formulation achieved the highest expansion ratio, whereas further GF addition produced smaller cells but higher foam density, demonstrating that cell refinement and foaming efficiency do not necessarily vary in parallel.

### 3.4. Mechanical Properties

**Figure 5:**
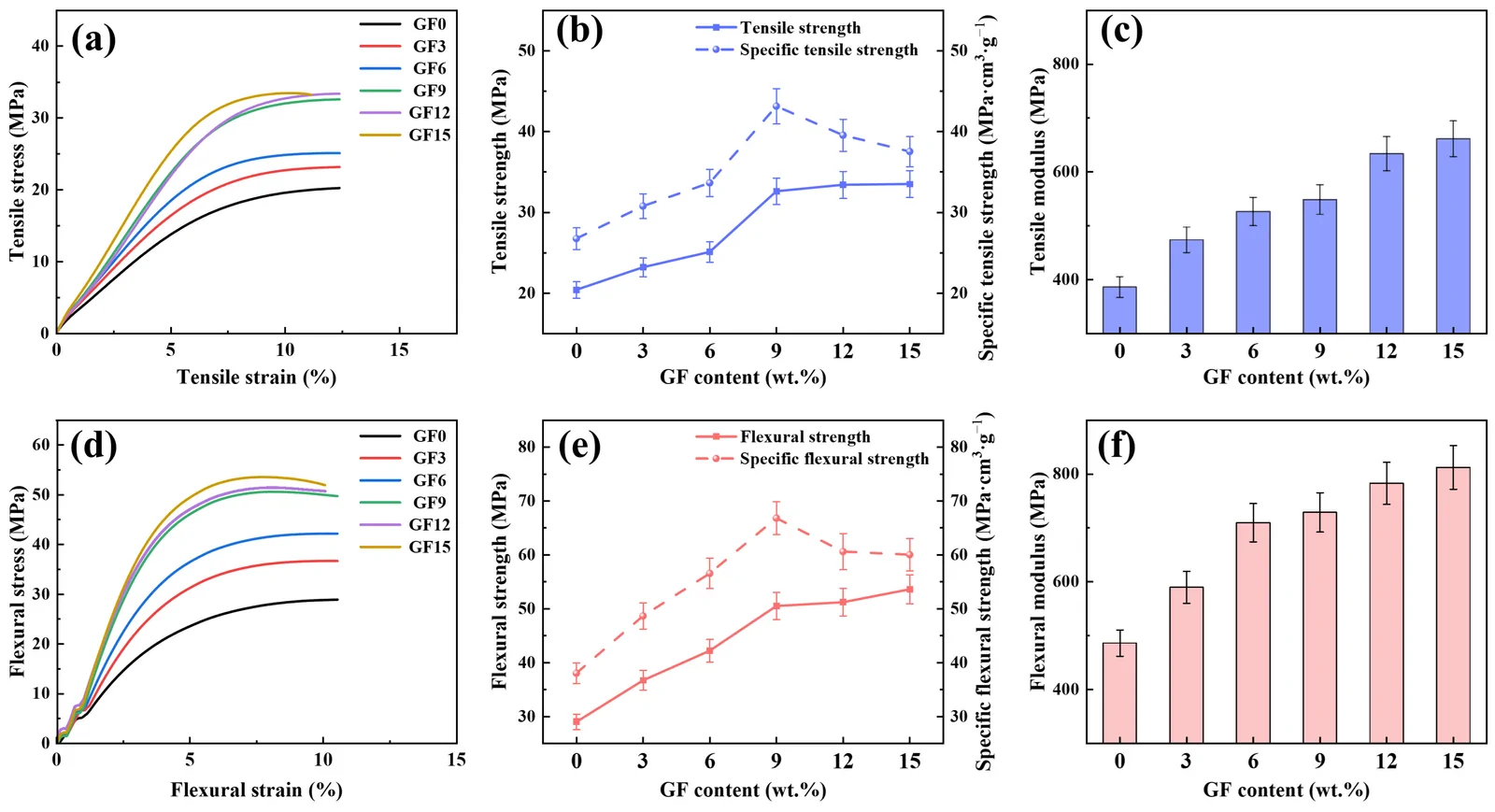
Tensile and flexural properties of GF/PP composite foams with different GF contents: (**a**) representative tensile stress–strain curves; (**b**) tensile strength and specific tensile strength; (**c**) tensile modulus; (**d**) representative flexural stress–strain curves; (**e**) flexural strength and specific flexural strength; and (**f**) flexural modulus. Error bars represent the standard deviations of ten valid measurements.

Figure 5 summarizes the tensile and flexural properties of the GF/PP composite foams. As the GF content increased from 0 to 15 wt.%, the tensile strength increased from 20.4 to 33.5 MPa and the tensile modulus increased from 386.1 to 661.2 MPa. Similarly, the flexural strength and modulus increased from 29.0 MPa and 486.2 MPa to 53.6 MPa and 812.3 MPa, respectively. These improvements are mainly attributed to the high strength and stiffness of GF and its contribution to load transfer within the composite [4,15,16,17].

Unlike the absolute strength and modulus, the specific tensile and flexural strengths initially increased and then decreased. Their highest measured values were obtained for GF9, reaching 43.13 MPa·cm^3^·g^−1^ and 66.81 MPa·cm^3^·g^−1^, respectively. At higher GF contents, the increase in foam density and the reduction in expansion ratio offset part of the reinforcing effect, thereby decreasing the load-bearing efficiency per unit mass. Thus, the continued increase in absolute strength did not result in a corresponding improvement in lightweight mechanical performance.

**Figure 6:**
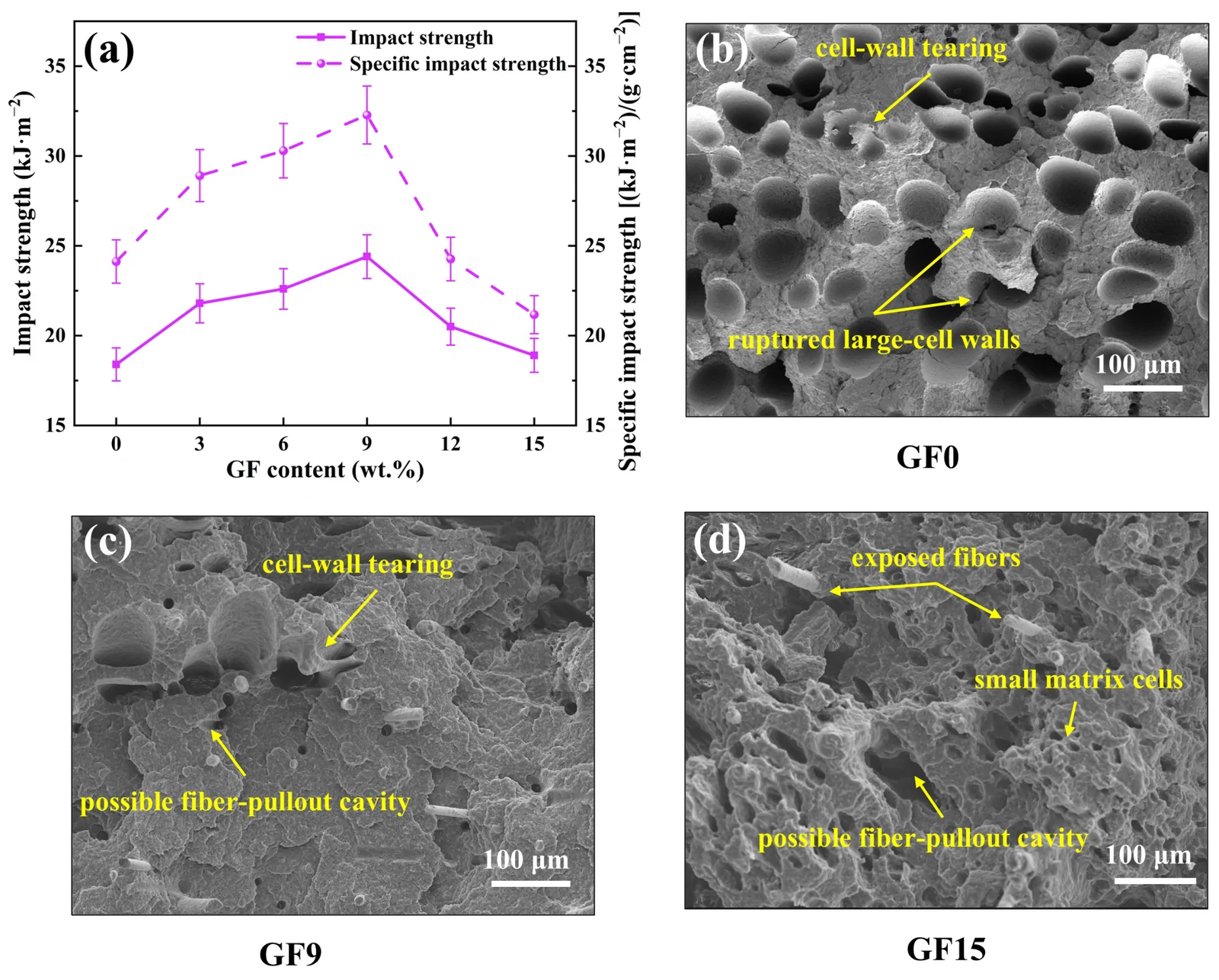
Impact properties and representative impact-fracture morphologies of GF/PP composite foams: (**a**) impact strength and specific impact strength as functions of GF content; and representative impact-fracture SEM micrographs of (**b**) GF0, (**c**) GF9, and (**d**) GF15. Error bars represent the standard deviations of ten valid measurements. The labeled fracture features are qualitative observations based on representative two-dimensional SEM images.

Figure 6 presents the impact properties and representative impact-fracture morphologies. The impact strength exhibited a nonmonotonic dependence on GF content and reached its highest measured mean value of 24.4 kJ·m^−2^ for GF9, approximately 32.6% higher than that of GF0. The corresponding specific impact strength was 32.28 kJ·m^−2^·cm^3^·g^−1^. The measured trend is consistent with the combined influence of cellular morphology and fiber-related deformation processes reported for fiber-reinforced microcellular composites [11,17,18,19]. When the GF content increased to 12 and 15 wt.%, however, the impact strength decreased to 20.5 and 18.9 kJ·m^−2^, respectively, indicating that further GF addition was unfavorable for impact toughness.

The fracture morphologies in Figure 6b–d are qualitatively consistent with the measured impact behavior. The representative GF0 fracture surface contained relatively large cells, with cracking and rupture visible along several cell walls. Such large cells may reduce the effective load-bearing area and increase local stress concentration, making the cell walls potential sites for crack initiation and propagation. In comparison, the representative GF9 fracture surface exhibited cell-wall tearing and fiber-adjacent cavities consistent with possible fiber pullout. These qualitative features may be associated with additional deformation and energy dissipation; however, they are not regarded as direct evidence of interfacial debonding or quantitatively verified fiber pullout. The representative GF15 fracture surface showed more exposed fibers and irregular fiber-adjacent void-like features, indicating greater local morphological heterogeneity. These features may promote local stress concentration and crack propagation, which is consistent with the decrease in the measured impact strength at high GF contents.

Increasing the GF content continuously improved the absolute tensile and flexural properties, but the specific properties and impact toughness did not increase monotonically. Based on the mean values, GF9 gave the best compromise between mechanical performance and density reduction among the tested formulations.

### 3.5. Structure–Property Relationships

**Figure 7:**
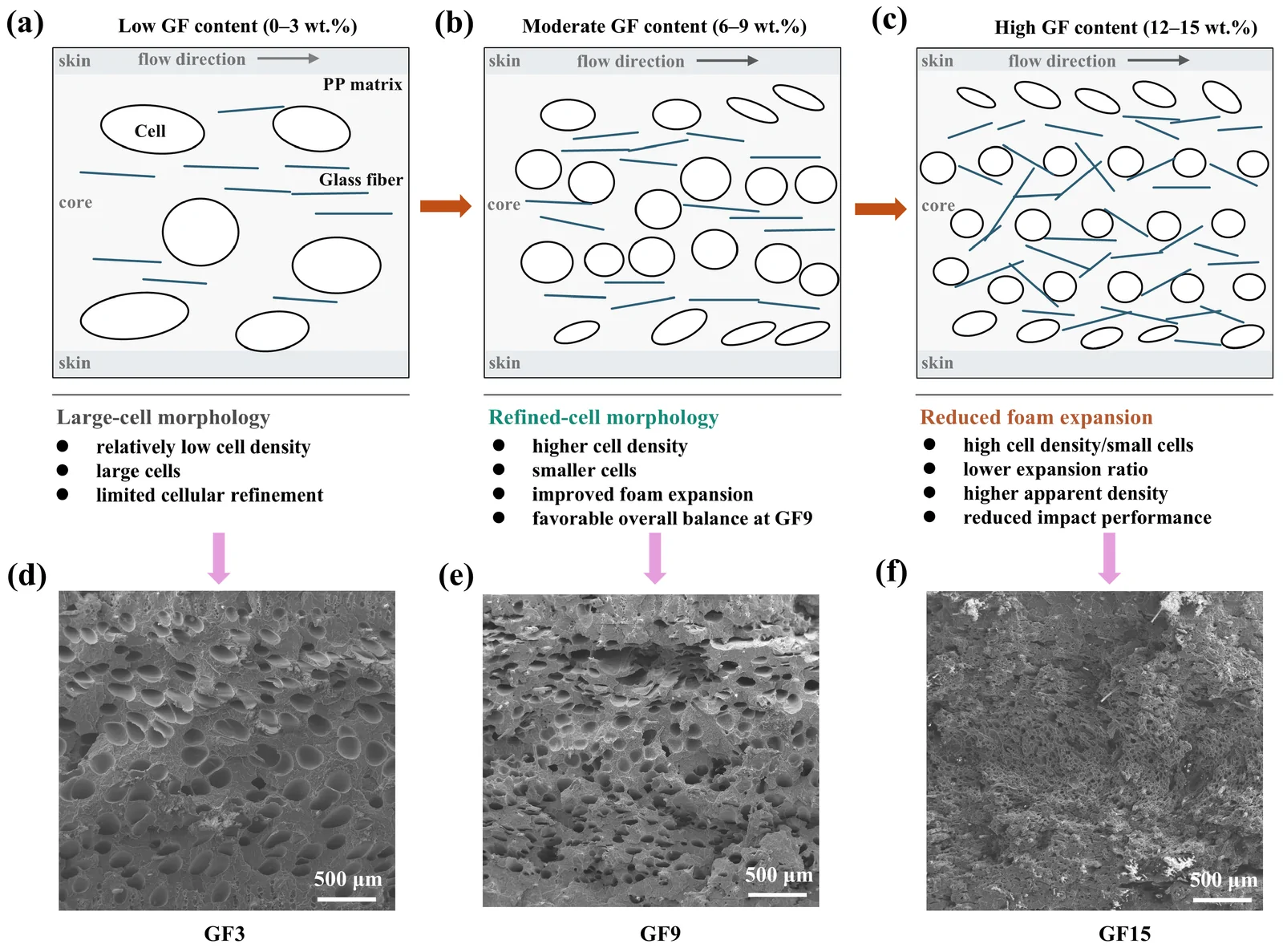
Conceptual illustration of the evolution of cellular morphology in GF/PP composite foams at (**a**) low GF content (0–3 wt.%), (**b**) moderate GF content (6–9 wt.%), and (**c**) high GF content (12–15 wt.%), together with representative SEM micrographs of (**d**) GF3, (**e**) GF9, and (**f**) GF15. The orange arrows indicate increasing GF content, while the pink arrows indicate the correspondence between the schematic morphologies and the representative SEM micrographs.

**Figure 8:**
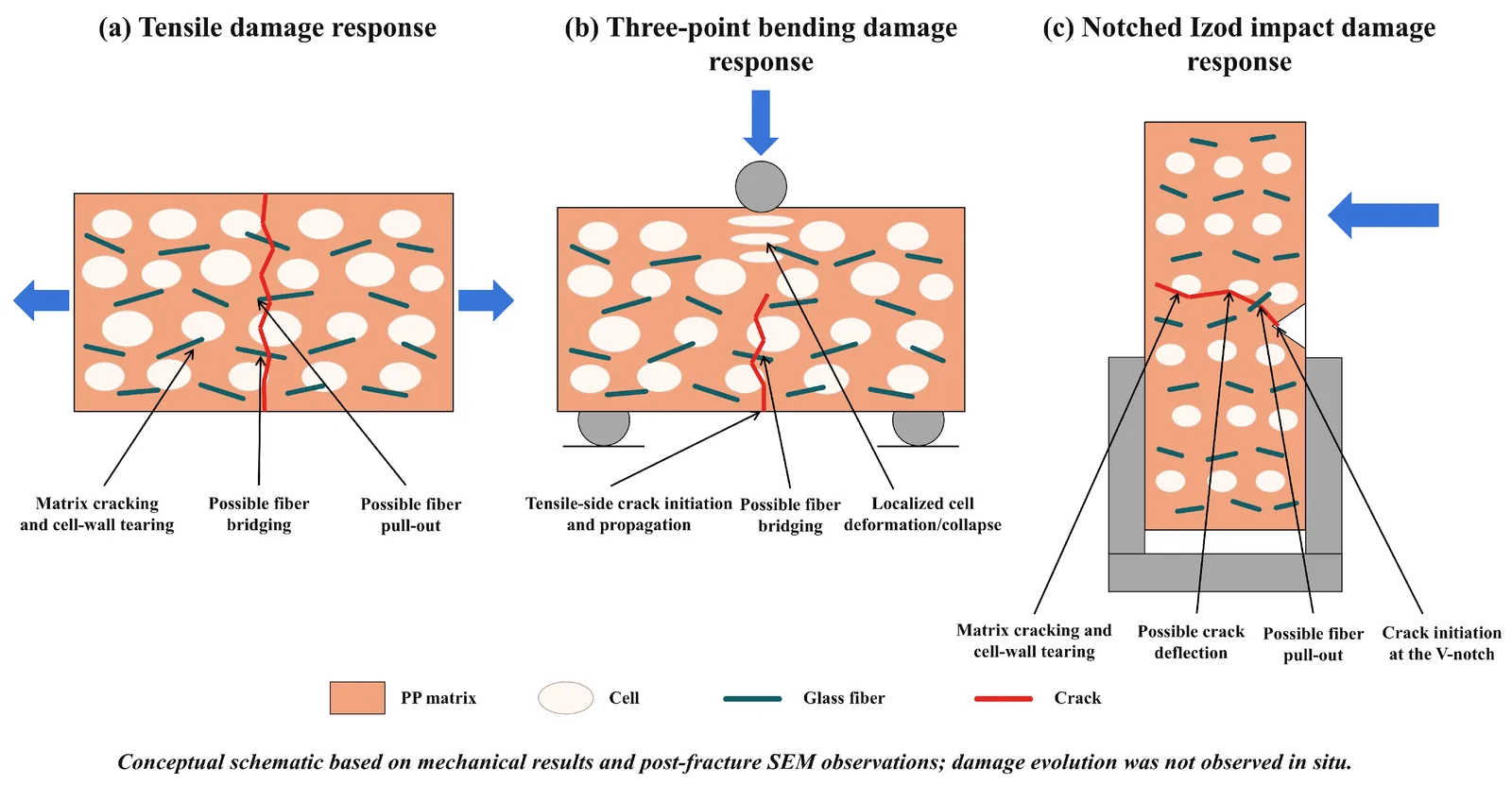
Conceptual schematics of the possible damage responses of GF/PP composite foams under (**a**) tensile loading, (**b**) three-point bending, and (**c**) notched Izod impact loading. The illustrated processes represent qualitative interpretations based on the mechanical results and representative post-fracture SEM morphologies rather than direct in situ observations. The blue arrows indicate the loading or impact directions, while the gray components represent the loading/supporting fixtures in the three-point bending test and the specimen-clamping fixture in the notched Izod impact test.

Figure 7 and Figure 8 summarize the changes in cellular morphology and mechanical response with GF content. As the GF content increased, the GF/PP composite foams exhibited a progressive increase in cell density and a decrease in average cell diameter. However, foam expansion and density-normalized mechanical performance varied nonmonotonically, indicating that cellular refinement alone did not necessarily produce greater weight reduction or better mechanical efficiency.

As illustrated in Figure 7, the foams containing 0–3 wt.% GF exhibited relatively low cell densities and large cells. At intermediate GF contents of 6–9 wt.%, the cells became smaller and more numerous, while the foam expansion was improved. GF6 exhibited the lowest apparent density of 0.746 g cm^−3^, whereas the expansion ratio reached its maximum value of 1.27 for GF9. When the mechanical results are considered together with density and expansion, GF9 gave the best overall result among the formulations tested.

When the GF content increased to 12–15 wt.%, the cell density continued to increase, and the average cell diameter continued to decrease. Nevertheless, the expansion ratios of GF12 and GF15 decreased to 1.16 and 1.12, respectively, while their apparent densities increased to 0.845 and 0.893 g cm^−3^. Thus, the formation of numerous small cells at high GF contents did not lead to greater foam expansion. The increased resistance of the composite melt to deformation and the greater geometric obstruction imposed by the fibers may have restricted cell expansion. In addition, because PP-g-MAH was fixed at 5 wt.%, the decreasing PP-g-MAH/GF ratio may also have influenced the interfacial environment at higher GF contents.

Figure 8 relates the observed structural evolution to the tensile, flexural, and impact responses. The tensile and flexural strengths and moduli generally increased with GF content, consistent with the reinforcing effect of the fibers and their resistance to matrix deformation. During tensile loading, matrix and cell-wall tearing, together with possible fiber-related processes such as bridging and pullout-related deformation, may contribute to the overall damage response. Under three-point bending, cracking is expected to develop primarily on the tensile side, whereas localized cell deformation or collapse may occur on the compression side. The proposed processes are schematic interpretations and were not observed in situ.

Impact performance was more sensitive to cellular morphology and local defects. GF9 exhibited the highest measured mean impact strength among the investigated formulations. Cell-wall tearing and fiber-adjacent cavities consistent with possible fiber pullout were observed in its representative fracture surface. These qualitative features may be associated with additional deformation and energy dissipation, although the representative two-dimensional SEM image does not provide direct evidence of fiber pullout or interfacial debonding. In comparison, more irregular fiber-adjacent void-like features and small matrix cells were observed in the representative GF15 fracture surface. These features may promote local stress concentration and provide preferential sites for crack propagation, which is consistent with the decrease in impact performance beyond GF9.

The results show a change from large cells and low cell density at low GF contents to a finer cellular structure at intermediate contents, followed by reduced foam expansion at high contents. Figure 7 and Figure 8 are schematic interpretations of the measured results; they do not constitute direct measurements of cell-nucleation kinetics, three-dimensional fiber orientation, or fiber networking.

## 4. Conclusions

GF/PP composite foams containing 0–15 wt.% GF were prepared by fixed-cavity MuCell injection molding with the PP-g-MAH content maintained at 5 wt.%. The effects of GF content on melt rheology, crystallization, cellular morphology, and lightweight mechanical performance were systematically investigated. Increasing GF content raised the apparent viscosity of the melts without dissolved gas and shifted crystallization toward higher temperatures. The peak crystallization temperature increased from 119.6 °C to a maximum of 126.9 °C, while the apparent crystallinity reached its maximum of 30.2% for GF6. From GF0 to GF15, the cell density increased from 6.12 × 10^4^ to 1.56 × 10^7^ cells cm^−3^, whereas the average cell diameter decreased from 264.5 to 27.1 μm. However, the expansion ratio reached its maximum of 1.27 for GF9 and subsequently decreased to 1.12 for GF15, demonstrating that cell refinement and foam expansion did not evolve synchronously with GF content.

The absolute tensile and flexural properties increased progressively with GF content. The tensile and flexural strengths increased from 20.4 and 29.0 MPa for GF0 to 33.5 and 53.6 MPa for GF15, respectively. In contrast, GF9 exhibited the highest measured mean density-normalized mechanical properties, with specific tensile, flexural, and impact strengths of 43.13 MPa·cm^3^·g^−1^, 66.81 MPa·cm^3^·g^−1^, and 32.28 kJ·m^−2^·cm^3^·g^−1^, respectively. Its mean impact strength reached 24.4 kJ·m^−2^, representing an increase of 32.6% relative to GF0. Among the formulations examined, GF9 therefore gave the best measured combination of cell refinement, foam expansion, fiber reinforcement, and weight-specific mechanical performance. The results also show that smaller cells alone do not guarantee better expansion or lightweight efficiency. The data support, but do not directly prove, competition between GF-assisted heterogeneous nucleation and restricted cell expansion at high GF content.

## Figures and Tables

**Table 1:** Comparison of processing conditions, cellular structures, and mechanical properties reported for GF/PP composite foams.

Ref.	GF Content (wt.%)	Process Conditions	Cell Size (μm)	Cell Density (Cells cm^−3^)	Representative Mechanical Properties
[10]	10.6, 17.9, 22.8; GF roving	MuCell injection molding	<30	NR	TS = 35.69 MPa; FS = 44.68 MPa; IS = 76.44 J m^−1^
[11]	20; short GF	MuCell injection molding	2–252	1.0 × 10^6^–5.5 × 10^6^	FS = 66.6 MPa; FM = 2888 MPa; IS = 18.5 kJ m^−2^
[12]	30; long GF	MuCell or Ku-Fizz injection molding with/without Core Back	5–248	6.6 × 10^5^–7.9 × 10^6^	TS = 65.5 ± 1.9 MPa; TM = 4623 ± 120 MPa; FS = 144.6 ± 2.2 MPa; FM = 6849 ± 98 MPa; IS = 25.7 ± 2.2 kJ m^−2^
[13]	0, 5, 10, 20; short GF	Mold-opening microcellular injection molding	32–153	9.04 × 10^7^–1.23 × 10^9^	FM = 1.69 GPa; TS = +59.5%; TM = +54.8%
Present study	0, 3, 6, 9, 12, 15; short GF	Fixed-cavity MuCell injection molding	27.1–264.5	6.12 × 10^4^–1.56 × 10^7^	TS = 20.4–33.5 MPa; FS = 29.0–53.6 MPa; IS_max_ = 24.4 kJ m^−2^ (GF9)

TS: tensile strength; TM: tensile modulus; FS: flexural strength; FM: flexural modulus; IS: impact strength; IS_max_: maximum impact strength; NR: not reported. For Ref. [10], the reported GF/PP mass ratios of 11.8%, 21.8%, and 29.6% were converted to GF mass fractions of 10.6, 17.9, and 22.8 wt.%, respectively, using *m*_GF_/(*m*_GF_ + *m*_PP_) × 100%. The mechanical properties were obtained using different specimen geometries, foam densities, sampling positions, and testing methods and should therefore not be used for direct quantitative ranking.

**Table 2:** Formulations of GF-reinforced PP composites with different GF contents.

Sample Number	GF (wt.%)	PP (wt.%)	PP-g-MAH (wt.%)
GF0	0	95	5
GF3	3	92	5
GF6	6	89	5
GF9	9	86	5
GF12	12	83	5
GF15	15	80	5

**Table 3:** DSC data for GF/PP composites with different GF contents.

Sample Number	*T*_c_ (°C)	*T*_onset_ (°C)	*T*_m_ (°C)	Δ*H*_m_ (J g^−1^)	*χ*_c_ (%)
GF0	119.6	125.0	175.9	57.9	27.7
GF3	121.0	126.2	176.3	60.2	29.7
GF6	122.5	128.0	176.8	59.4	30.2
GF9	124.2	128.9	174.7	54.2	28.5
GF12	126.9	132.8	173.9	50.5	27.5
GF15	125.9	131.4	173.6	48.3	27.2

Here, *T*_onset_ denotes the onset crystallization temperature; *T*_c_ denotes the peak crystallization temperature; *T*_m_ denotes the melting temperature; Δ*H*_m_ denotes the melting enthalpy; and *χ*_c_ denotes the apparent crystallinity of the PP-based polymer phase.

## Data Availability

The original contributions presented in this study are included in the article. Further inquiries can be directed to the corresponding author.
